# 

**Published:** 1881-06

**Authors:** 


### CONTENTS:

ORIGINAL LECTURES:

I. Inaugural Address at the opening of the Woman's Medical College of the New York Infirmary. By M. Putnam Jacobi, M.D. .... 561

II. Pathology and Treatment of Yellow Fever; with Some Remarks upon the Nature of its Cause and its Prevention. By H. D. Schmidt, M.D. .... 585

III. Some Points in Diphtheria. By A. T. Conley, M.D. .... 622

SOCIETY REPORTS:

IV. Chicago Biological Society .... 626

V. Chicago Medical Society .... 630

VI. West Chicago Medical Society .... 634

FOREIGN CORRESPONDENCE:

VII. Vienna Letter .... 636

DOMESTIC CORRESPONDENCE:

VIII. New York Letter .... 638

EDITORIAL .... 644

REVIEWS AND BOOK NOTICES .... 645

SUMMARY:

*Quarterly Report of Ophthalmological and Otological Literature* .... 652

*Practical Medicine* .... 658

SELECTIONS:

The Physician's Leisure; a Plea for the Study of Archæology .... 660

ITEMS .... 625, 659, 671

ANNOUNCEMENTS FOR THE MONTH .... 672

Notice to Correspondents ....

AMERICAN MEDICAL ASSOCIATION.—Proceedings of Thirty-second Annual Session .... Supplement.

